# Supplementary material for: Glutamine deficiency in solid tumor cells confers resistance to ribosomal RNA synthesis inhibitors
Source: Nat Commun. 2022 Jun 28;13:3706. doi: 10.1038/s41467-022-31418-w (PMC9240073; doi:10.1038/s41467-022-31418-w)
Supplement: Supplementary file 1 — Supplementary Information [file 41467_2022_31418_MOESM1_ESM.pdf]

Glutamine deficiency in solid tumor cells confers resistance to ribosomal RNA synthesis inhibitors

Melvin Pan et al.

a

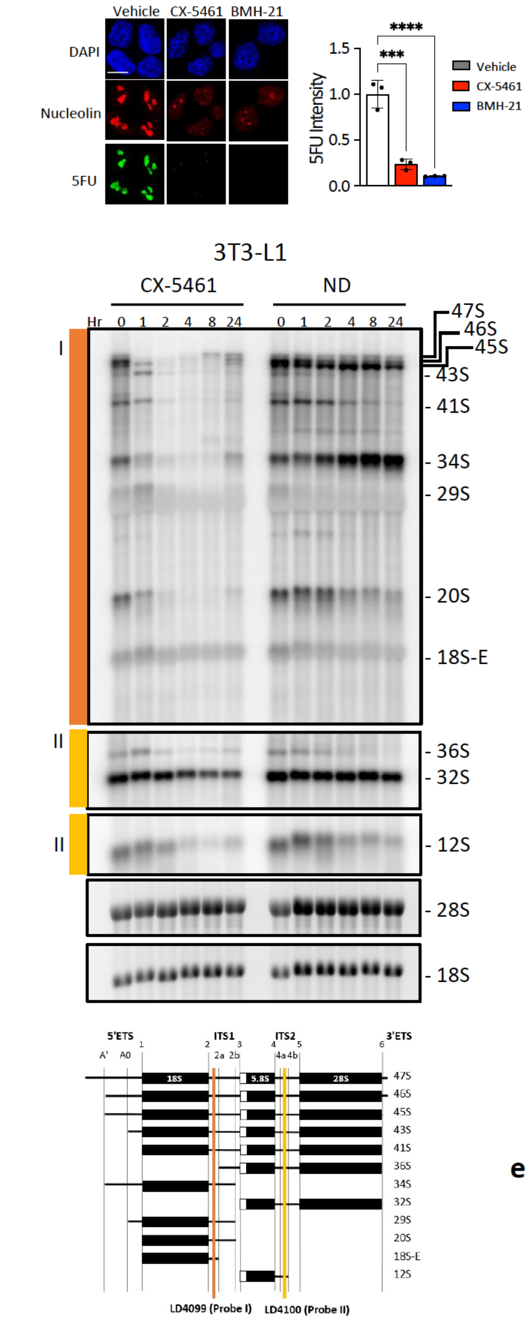

b

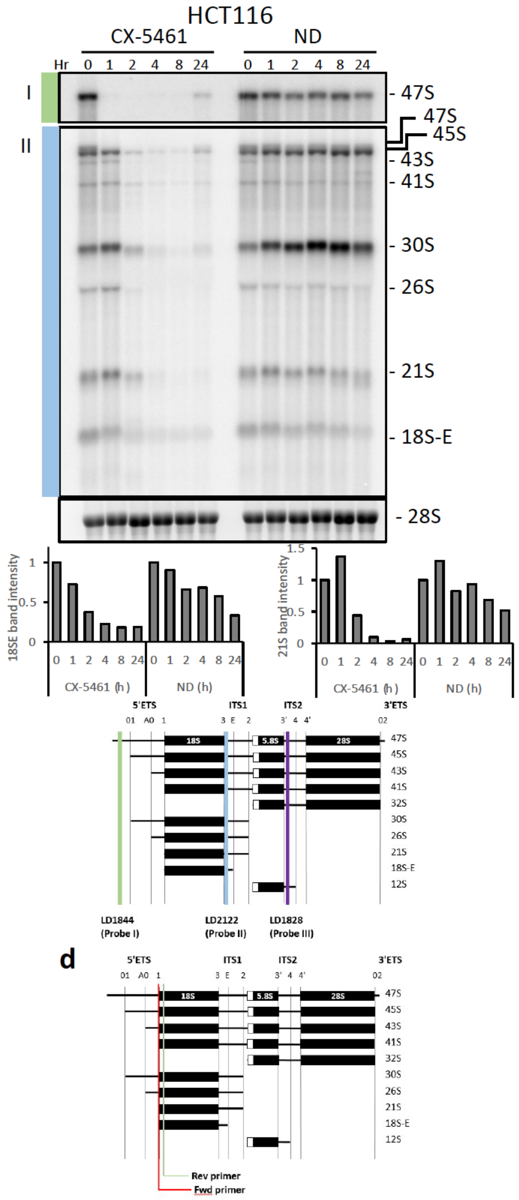

e

HCT116 Tumor	Treatment	Replicate	Actinomycin D (mM)
Periphery	Control	1	Not detected
		2	Not detected
Core	Control	1	Not detected
		2	Not detected
		3	Not detected
Periphery	ActD injection	1	2.20
		2	0.90
		3	2.10
Core	ActD injection	1	0.80
		2	1.00
		3	0.10

Supplementary Fig. 1

(a) CX-5461 and BMH-21 inhibits 5-Fluorouridine (5FU) uptake in HCT116 cells. HCT116 cells were treated to 10 µM CX-5461 or 1 µM BMH-21 for 1 hr, and nascent RNA was pulse-labeled with 5FU for 5 minutes. Data represent mean ± SD of $n = 3$ independent images. *P*-values (left to right): < 0.0001, < 0.0001 (****p < 0.0001, statistical analysis by one-way ANOVA). Scale bar, 10 µm.

(b) Top: Pre-rRNA expression in HCT116 cells treated to 10 μM CX-5461 or ND as assessed using northern blotting. Middle: Quantification of 21S and 18S-E species in response to CX-5461 or ND treatment. Bottom: binding sites of northern blot probes.
(c) Top: Pre-rRNA expression in mouse 3T3-L1 cells treated to 10 μM CX-5461 or ND as assessed using northern blotting. Bottom: binding sites of northern blot probes.
(d) Binding sites of the 5'ETS pre-rRNA qRT-PCR primers. The forward primer recognizes site 01 of the pre-rRNA, which is cleaved upon maturation. Mature rRNAs do not contain the site 01 sequence.
(e) Actinomycin D concentration in HCT116 tumor tissues following intratumoral injection of actinomycin D.

a

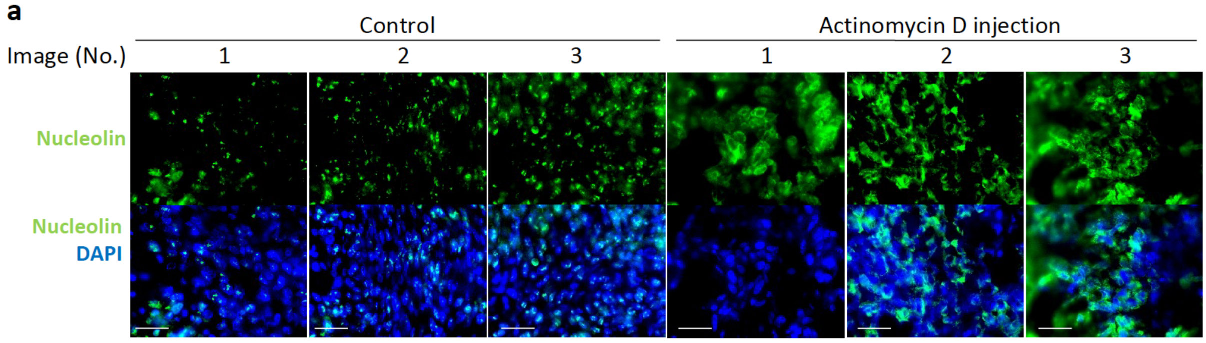

b

Compound	Periphery (nmol/gram)	Core (nmol/gram)	Periphery (normalized to 1)	Core (relative to periphery)	p-value
Urocanate	602.81	14.07	1.0	0.023	0.008991784
Histamine	195.42	20.17	1.0	0.103	0.026806533
5-Oxoproline	937.97	138.45	1.0	0.148	0.01342651
Arg	751.84	139.44	1.0	0.185	0.0012917
Cytidine	9.86	2.69	1.0	0.273	0.010154887
Glutathione(red)	2533.56	744.07	1.0	0.294	0.000529169
Ser	2629.26	793.44	1.0	0.302	0.003870969
Anserine	7.06	2.33	1.0	0.330	0.032765351
IMP	563.22	188.46	1.0	0.335	0.014767429
Argininosuccinate	28.64	10.18	1.0	0.356	0.000503562
UDP	48.81	17.70	1.0	0.363	0.005865504
Glycerophosphate	238.82	88.59	1.0	0.371	0.000424676
UMP	579.36	218.28	1.0	0.377	0.00149572
CDP	11.04	4.43	1.0	0.401	0.001914604
AMP	609.28	259.65	1.0	0.426	0.011314459
Carnosine	8.65	3.74	1.0	0.432	0.000259768
Cystathionine	10.80	4.68	1.0	0.433	0.022993214
CMP	87.89	38.07	1.0	0.433	1.96385E-05
UDP-glucose	209.63	90.85	1.0	0.433	0.001532262
GMP	286.98	127.93	1.0	0.446	0.002287422
Glycerophosphorylcholine	61.40	27.61	1.0	0.450	0.002795165
gamma-Glu-cys	5.86	2.63	1.0	0.450	0.009944522
UDP-glucuronate	72.42	32.63	1.0	0.451	0.00030003
Hypotaurine	233.51	106.61	1.0	0.457	0.006931956
GDP-mannose	5.04	2.31	1.0	0.458	0.033945489
Gln	3152.53	1475.04	1.0	0.468	2.78503E-06
Spermidine	21.77	10.40	1.0	0.478	0.000682741
UTP	5.39	2.58	1.0	0.480	0.020690314
UDP-N-acetylglucosamine	479.11	236.26	1.0	0.493	0.000413227
His	311.53	159.12	1.0	0.511	0.005440344
Val	801.58	409.45	1.0	0.511	0.003877367
dTMP	9.64	4.94	1.0	0.512	0.001589071
Trehalose 6-phosphate	62.13	31.99	1.0	0.515	0.002041459
Asn	589.84	307.43	1.0	0.521	5.23679E-05
Ru5P	54.98	29.25	1.0	0.532	0.009278918
NADH	5.41	2.90	1.0	0.536	0.01348221
Phe	357.79	194.47	1.0	0.544	0.003501808
SAM+	26.04	14.55	1.0	0.559	0.001199224
ADP	170.88	95.46	1.0	0.559	0.012560832
beta-Ala	138.47	79.41	1.0	0.573	0.000314044
5-Aminolevulinate	1.54	0.89	1.0	0.575	0.030835195
G1P	22.63	13.03	1.0	0.576	0.013847607
N8-Acetylspermidine	6.69	3.87	1.0	0.578	0.025265653
N-Acetylaspartate	241.99	139.98	1.0	0.578	0.005764296
CDP-choline	45.01	26.08	1.0	0.579	0.000593962
2AB	27.33	15.95	1.0	0.584	0.041846706
Met	235.94	137.88	1.0	0.584	0.003469603
Tyr	388.58	228.06	1.0	0.587	0.001016928
CMP-N-acetylneuraminate	16.36	9.75	1.0	0.596	0.001850367
N1,N12-Diacetylspermine	11.54	20.00	1.0	1.733	0.013976182

Supplementary Fig. 2
(a) Immunofluorescence staining of nucleolin in HCT116 tumors. HCT116 tumors were intratumorally injected with actinomycin D (1.33 mM) and were collected after 30 min for cryosectioning and immunofluorescence. Three representative images are shown per treatment. Scale bar, 50 µm.
(b) Metabolome analysis of HCT116 periphery and core tissues (n = 2 independent tumors x 2

technical replicates). Twenty metabolites (black text) were downregulated in core tissues, and three metabolites (red text) were upregulated. Data shows the mean. Two-tailed student's t-test was used. Metabolomics data is available in Source Data.

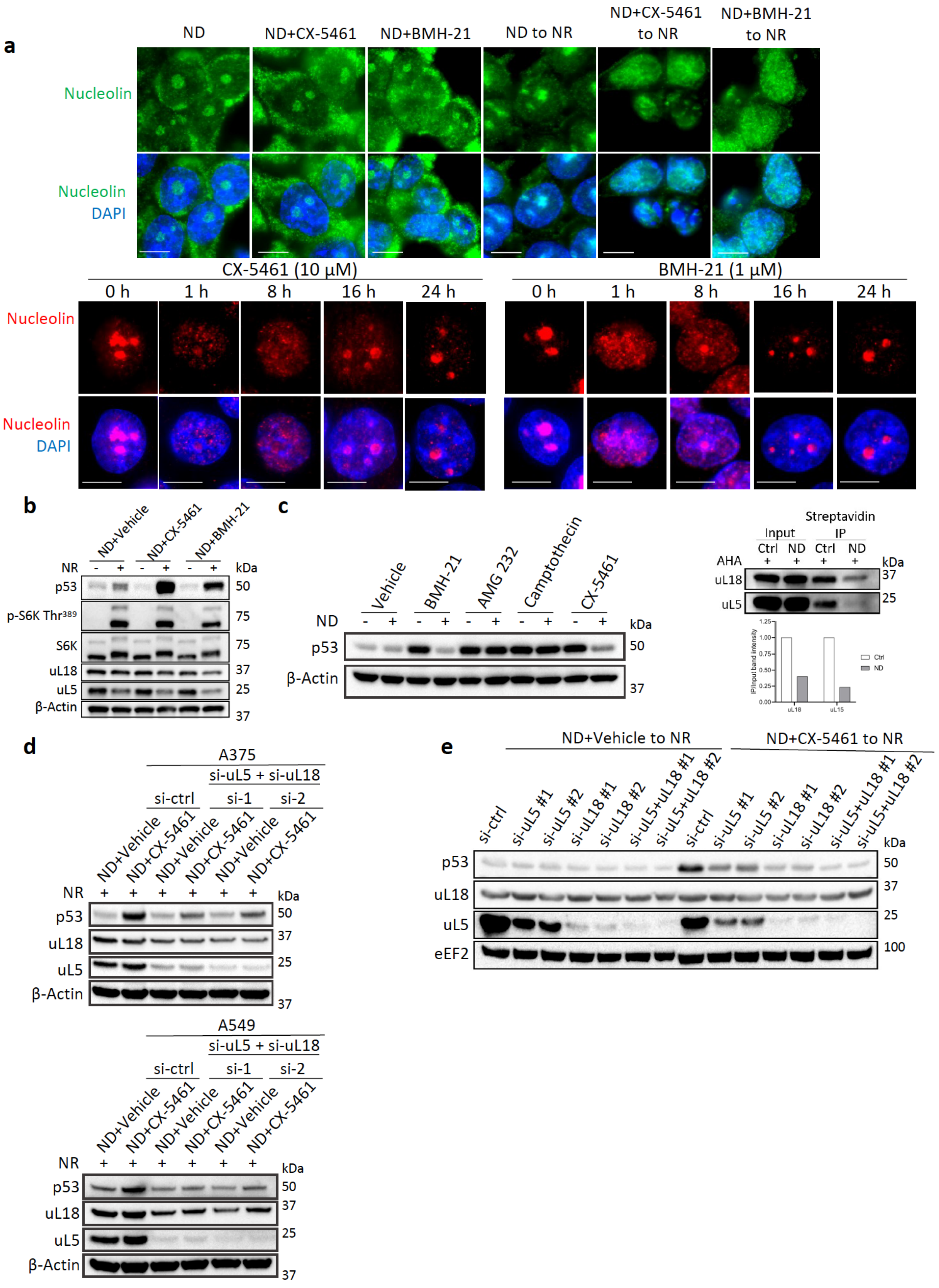

Supplementary Fig. 3

(a) Top: Nucleolin immunofluorescence on HCT116 cells treated to ND with vehicle (NaH_2PO_4), CX-5461 (10 μM), or BMH-21 (1 μM) for 24 h. After 24 h, cells were exposed to NR for 1 h. Bottom: HCT116 cells were treated to CX-5461 or BMH-21 over a time course of 24 h. This result showed that CX-5461/BMH-21 treatment transiently disrupts nucleolin localization, and that nucleoli begin to reform after 16 hours of drug treatment. Scale bar, 10 μm.

(b) NR induces p53 in pre-rRNA depleted A375 cells. A375 cells were cultured under ND with vehicle (NaH_2PO_4), CX-5461 (10 μM), or BMH-21 (1 μM) for 24 h; after 24 h, the treatment medium was replaced with a drug-free basal medium (NR) for 8 h.
(c) Left: CX-5461 (10 μM) or BMH-21 (1 μM) treatment does not activate p53 under ND, while AMG 232 (non-genotoxic MDM2 inhibitor) or camptothecin (topoisomerase inhibitor) stabilizes p53 regardless of nutrient availability. Right: Expression of newly translated uL5/uL18 under ND. Newly synthesized proteins were labeled with L-azidohomoalanine (AHA) and affinity purified using Streptavidin beads (Methods).
(d) Co-knockdown of uL5 and uL18 by siRNA decreases NR-mediated p53 activation. A375 and A549 cells were transfected with control or two different sets of siRNAs targeting uL5 and uL18. After 48 h, the cells were placed under ND with CX-5461 (10 μM) for 24 h. After 24 h, the treatment medium was replaced with DMEM for 8 h and the protein lysate was collected for western blotting.
(e) Independently silencing uL5 or uL18 inhibits NR-mediated p53 activation. HCT116 cells were transfected with control or uL5/uL18 siRNAs. After 48 h, the cells were placed under ND with CX-5461 (10 μM) for 24 h. After 24 h, the treatment medium was replaced with DMEM for 8 h and the protein lysate was collected for western blotting.

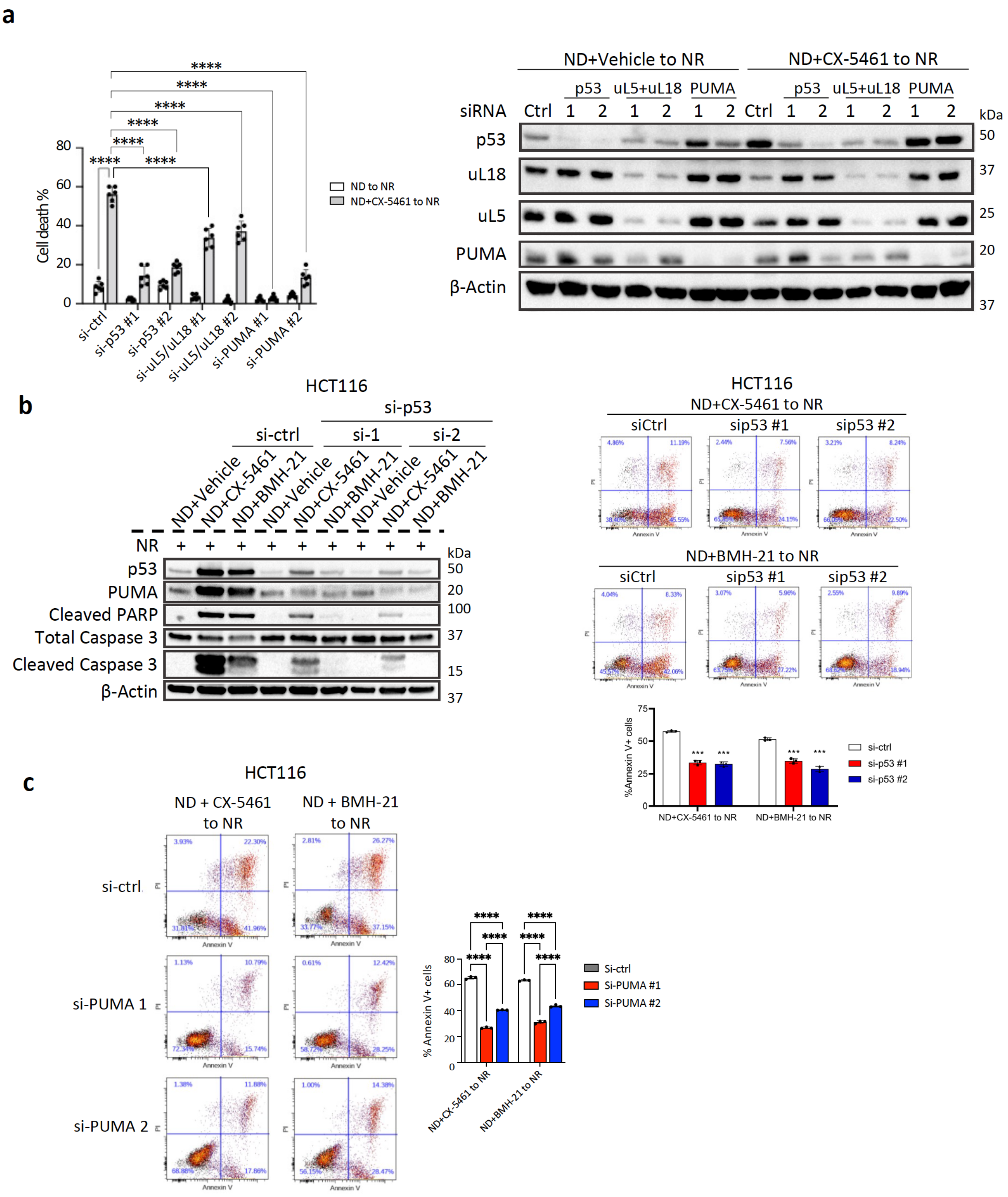

Supplementary Fig. 4

(a) Silencing uL5/uL18, p53, or PUMA, decreases NR-mediated cell death. HCT116 cells were transfected with siRNAs targeting uL5+uL18, p53, or PUMA. After 48 h, the cells were placed under ND with vehicle or CX-5461 (10 μM) for 24 h. After 24 h, the treatment medium was replaced with DMEM for 8 h and the protein lysate was collected for western blotting. Left: Percentage of cell death as assessed using the trypan blue exclusion assay. Right: Expression of p53, uL18, uL5, and PUMA as assessed using western blotting. Data represent mean ± SD of *n* = 6 technical replicates. *P*-values (left to right): < 0.0001, < 0.0001, < 0.0001, < 0.0001, < 0.0001, < 0.0001, < 0.0001 (****p < 0.0001, statistical analysis by two-way ANOVA).

(b) Silencing p53 decreases NR-mediated apoptosis in HCT116 cells. HCT116 were transfected

with scramble or two independent p53 siRNAs. After 48 h, cells were placed under ND with vehicle (NaH_2PO_4), CX-5461 (10 μM), or BMH-21 (1 μM). After 24 h, the treatment medium was replaced with a drug-free basal medium for 24 h. Left: Expression of cleaved caspase 3 and PARP as assessed using western blotting. Right: Percentage of Annexin V+ cells as assessed using flow cytometry. Data represent mean ± SD of *n* = 3 technical replicates. *P*-values (left to right): 1.3×10^{-5}, 9.6×10^{-6}, 8.4×10^{-5}, 3.9×10^{-5} (*$p < 0.05$; **$p < 0.01$; ***$p < 0.001$).

(c) PUMA siRNA knockdown inhibits NR-mediated apoptosis in pre-rRNA depleted HCT116 cells, as determined using Annexin V-PI flow cytometry. HCT116 were transfected with scramble or two independent PUMA siRNAs. After 48 h, cells were placed under ND with vehicle (NaH_2PO_4), CX-5461 (10 μM), or BMH-21 (1 μM). After 24 h, the treatment medium was replaced with a drug-free basal medium and Annexin V-PI flow cytometry was performed. Right: quantification of apoptosis (Annexin V+ cells) for the indicated conditions. Data represent mean ± SD of *n* = 3 technical replicates. *P*-values (left to right): < 0.0001, < 0.0001, < 0.0001, < 0.0001, < 0.0001, < 0.0001 (****$p < 0.0001$, statistical analysis by two-way ANOVA).

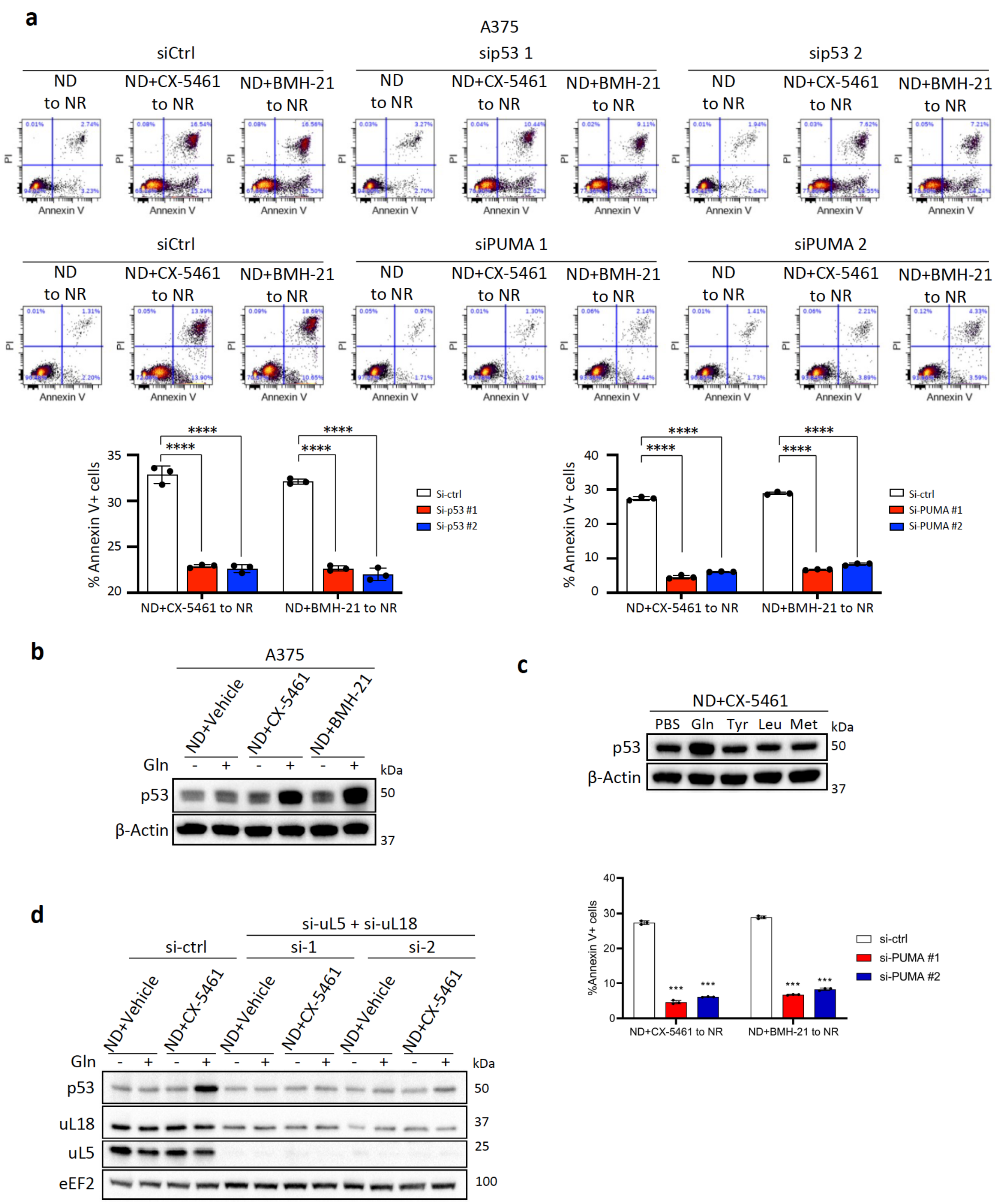

Supplementary Fig. 5

(a) p53 or PUMA knockdown by siRNA inhibits NR-mediated apoptosis in A375 cells, as assessed by Annexin V-PI flow cytometry. A375 were transfected with siRNAs, and after 48 h, cells were placed under ND with vehicle (NaH_2PO_4), CX-5461 (10 μM), or BMH-21 (1 μM). After 24 h, the treatment medium was replaced with a drug-free basal medium and flow cytometry was performed after 24 h NR. Data represent mean ± SD of $n = 3$ technical replicates. *P*-values (left to right): si-p53 experiment, < 0.0001, < 0.0001, < 0.0001, < 0.0001, si-PUMA experiment, < 0.0001 , < 0.0001, < 0.0001, < 0.0001 (****p < 0.0001, statistical analysis by two-way ANOVA).

(b) Glutamine (gln) activates p53 in pre-rRNA depleted A375 cells as assessed by western blotting. A375 were cultured in ND medium with vehicle (NaH_2PO_4), CX-5461 (10 µM), or BMH-21 (1 µM) for 24 h. After 24 h the old treatment media was replaced with ND media containing PBS or Gln (4 mM) for 8 h.
(c) Glutamine (gln) but not tyrosine (tyr), leucine (leu), or methionine (met) activates p53 in pre-rRNA deficient HCT116 cells. HCT116 cells were placed under ND with CX-5461 (10 µM) for 24 h. After 24 h the old treatment media was replaced with ND media containing PBS, glutamine (4 mM), tyrosine (0.1 mg/mL), leucine (0.1 mg/mL), or methionine (0.03 mg/mL). The concentration of amino acids added back to the ND media is equal to the concentrations in the basal media.
(d) Dual siRNA knockdown of uL5 and uL18 inhibits glutamine (Gln)-mediated p53 activation. HCT116 cells were transfected with control or two different sets of siRNAs targeting RPL5 and RPL11. After 48 h, cells were placed under ND with CX-5461 (10 µM) for 24 h. After 24 h the old treatment media was replaced with ND media containing PBS or Gln (4 mM) for 8 h.

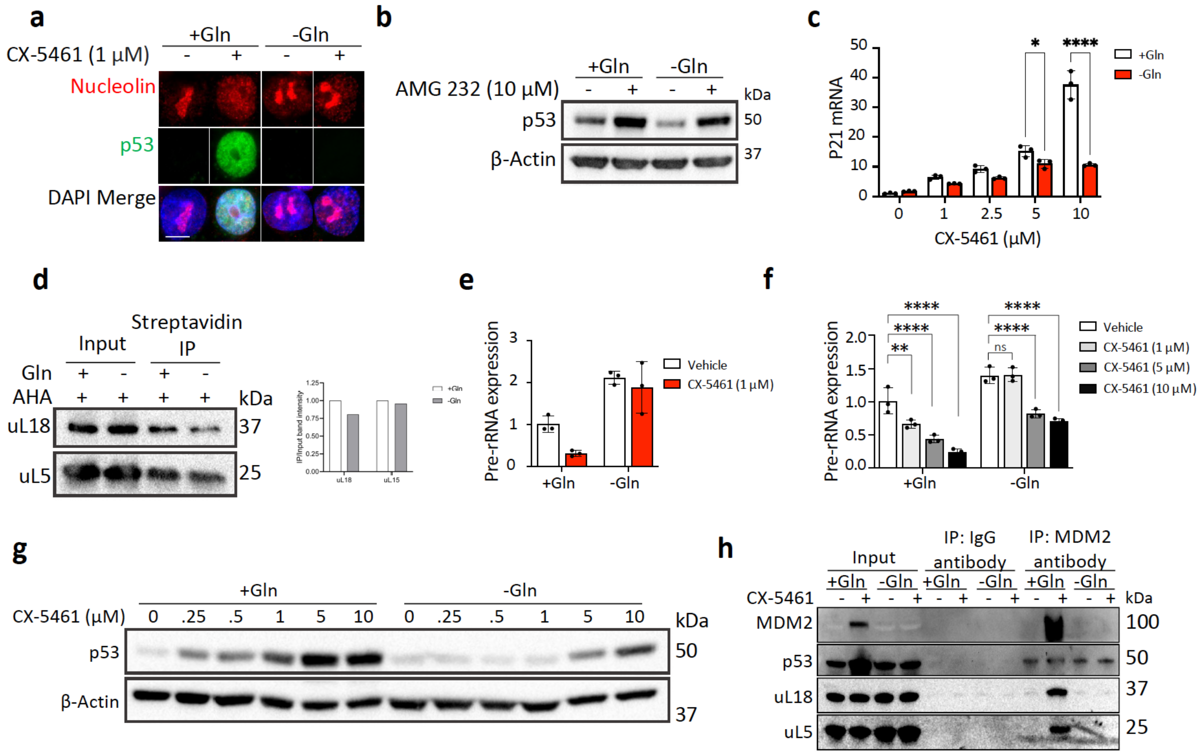

Supplementary Fig. 6

(a) p53 nuclear localization after CX-5461 (10 μM) treatment under glutamine replete or deprivation conditions. Scale bar, 10 μm.

(b) p53 expression after AMG 232 (10 μM) treatment under glutamine deprivation

(c) Glutamine deprivation inhibited p21 mRNA induction by CX-5461. HCT116 cells were incubated in glutamine replete (+Gln) or glutamine deprivation (-Gln) medium in the presence of vehicle or CX-5461 at the indicated concentrations for 24 h. Data represent mean ± SD of $n = 3$ technical replicates. *P*-values (left to right): 0.03990, < 0.0001 (*p < 0.05; ****p < 0.0001, statistical analysis by two-way ANOVA).

(d) Expression of newly synthesized uL5/uL18 under glutamine deprivation

(e) Pre-rRNA expression after CX-5461 treatment under glutamine deprivation. Data represent mean ± SD of $n = 3$ technical replicates.

(f) 10 μM CX-5461, but not 1 μM, inhibits pre-rRNA expression under glutamine deprivation. Data represent mean ± SD of $n = 3$ technical replicates. *P*-values (left to right): 0.0019, < 0.0001, < 0.0001, < 0.0001. (ns not significant, **p < 0.01; ****p < 0.0001, statistical analysis by two-way ANOVA).

(g) 10 μM CX-5461, but not 1 μM, activates p53 under glutamine deprivation.

(h) Expression of co-immunoprecipitated uL5/uL18 after MDM2 immunoprecipitation.

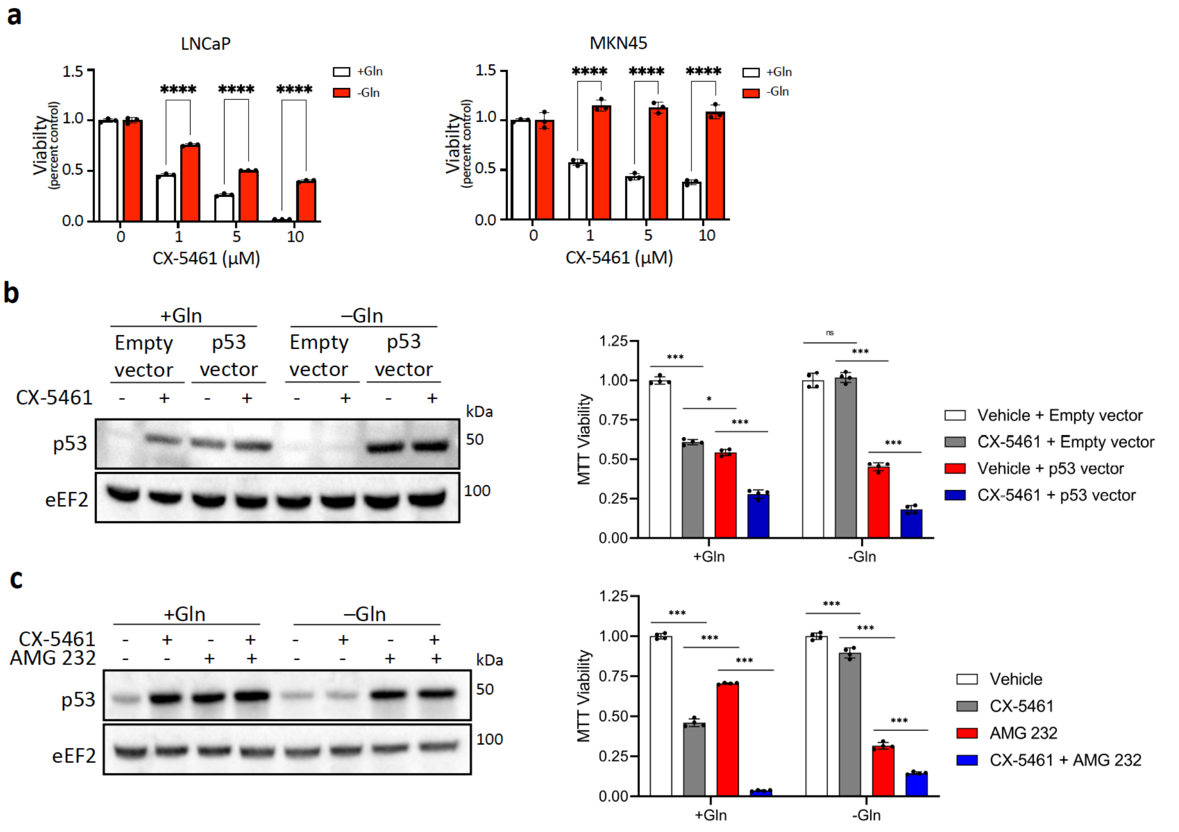

Supplementary Fig. 7

(a) Glutamine deprivation confers MKN45 and LNCaP cells resistance to CX-5461 as assessed by MTT assay. Data represent mean ± SD of $n = 3$ biological replicates. *P*-values (left to right): LNCaP, < 0.0001, < 0.0001, < 0.0001, MKN45, < 0.0001, < 0.0001, < 0.0001 (****p < 0.0001, statistical analysis by two-way ANOVA).

(b) p53 overexpression decreases cell viability under glutamine deprivation. HCT116 cells were transfected with empty vector (pcDNA3.1) or p53 vector. After 24 h, the transfected cells were cultured in glutamine replete (+Gln) or glutamine deprived (-Gln) media with vehicle or 1 µM CX-5461 added to the metabolic media. Cell viability was assessed 48 h later using the MTT assay. Data represent mean ± SD of $n = 4$ biological replicates. *P*-values (top to bottom): +Gln, 8.0×10^{-8}, 0.0011, 2.0×10^{-6}, -Gln, 0.27, 7.1×10^{-8}, 2.5×10^{-6} (ns, not significant; *p < 0.05; **p < 0.01; ***p < 0.001).

(c) Induction of p53 with AMG 232 decreases cell viability under glutamine deprivation. HCT116 cells were cultured in glutamine replete (+Gln) or glutamine deprived (-Gln) media with a combination of 1 µM CX-5461 and 1 µM AMG 232. Cell viability was assessed 72 h later using the MTT assay. Data represent mean ± SD of $n = 4$ biological replicates. *P*-values (top to bottom): +Gln, 1.4×10^{-8}, 5.14×10^{-7}, 2.7×10^{-13}, -Gln, 7.5×10^{-4}, 4.3×10^{-8}, 2.6×10^{-6} (*p < 0.05; **p < 0.01; ***p < 0.001).
