# Supplementary material for: Glutamine deficiency in solid tumor cells confers resistance to ribosomal RNA synthesis inhibitors
Source: Nat Commun. 2022 Jun 28;13:3706. doi: 10.1038/s41467-022-31418-w (PMC9240073; doi:10.1038/s41467-022-31418-w)
Supplement: Supplementary file 2 — Reporting Summary [file 41467_2022_31418_MOESM2_ESM.pdf]

nature research

Corresponding author(s): Tsuyoshi Osawa

Last updated by author(s): Mar 22 2022

Reporting Summary

Nature Research wishes to improve the reproducibility of the work that we publish. This form provides structure for consistency and transparency in reporting. For further information on Nature Research policies, see our Editorial Policies and the Editorial Policy Checklist.

Statistics

For all statistical analyses, confirm that the following items are present in the figure legend, table legend, main text, or Methods section.

n/a	Confirmed	
☐	☒	The exact sample size (*n*) for each experimental group/condition, given as a discrete number and unit of measurement
☐	☒	A statement on whether measurements were taken from distinct samples or whether the same sample was measured repeatedly
☐	☒	The statistical test(s) used AND whether they are one- or two-sided<br>*Only common tests should be described solely by name; describe more complex techniques in the Methods section.*
☒	☐	A description of all covariates tested
☒	☐	A description of any assumptions or corrections, such as tests of normality and adjustment for multiple comparisons
☐	☒	A full description of the statistical parameters including central tendency (e.g. means) or other basic estimates (e.g. regression coefficient) AND variation (e.g. standard deviation) or associated estimates of uncertainty (e.g. confidence intervals)
☐	☒	For null hypothesis testing, the test statistic (e.g. *F*, *t*, *r*) with confidence intervals, effect sizes, degrees of freedom and *P* value noted<br>*Give P values as exact values whenever suitable.*
☒	☐	For Bayesian analysis, information on the choice of priors and Markov chain Monte Carlo settings
☒	☐	For hierarchical and complex designs, identification of the appropriate level for tests and full reporting of outcomes
☒	☐	Estimates of effect sizes (e.g. Cohen's *d*, Pearson's *r*), indicating how they were calculated

Our web collection on statistics for biologists contains articles on many of the points above.

Software and code

Policy information about availability of computer code

Data collection	No software was used
Data analysis	No software was used

For manuscripts utilizing custom algorithms or software that are central to the research but not yet described in published literature, software must be made available to editors and reviewers. We strongly encourage code deposition in a community repository (e.g. GitHub). See the Nature Research guidelines for submitting code & software for further information.

Data

Policy information about availability of data

All manuscripts must include a data availability statement. This statement should provide the following information, where applicable:

- Accession codes, unique identifiers, or web links for publicly available datasets
- A list of figures that have associated raw data
- A description of any restrictions on data availability

We have provided a complete data availability statement under a "data availability" section in the manuscript. Further, the minimal dataset required to replicate experiments are outlined in the methods section.

Field-specific reporting

Please select the one below that is the best fit for your research. If you are not sure, read the appropriate sections before making your selection.

☒ Life sciences ☐ Behavioural & social sciences ☐ Ecological, evolutionary & environmental sciences

For a reference copy of the document with all sections, see nature.com/documents/nr-reporting-summary-flat.pdf

Life sciences study design

All studies must disclose on these points even when the disclosure is negative.

Sample size

Sample sized was based off of previous studies that performed similar experiments. For MTT assays, sample size (biological replicates) were based off of Delaidelli et al (PMID 28574509). For the xenograft studies (n = 8 mice per treatment group), this sample size was based off of Drygin et al (PMID 21159662). For immunofluoresence quantification experiments, the sample size (number of images taken) was based off of Somasekharan et al (PMID 25800057).

Data exclusions

No data were excluded from the analysis

Replication

All findings (except Fig 2A, the labor intensive pulse-chase time course experiment) were replicated using two independent experiments. Major findings (ie western blots showing glutamine deprivation inhibits p53 activation) were reproduced at least four times in independent experiments.

Randomization

For tumor growth experiments, tumor bearing mice were randomly placed into vehicle, CX-5461, or AMG-232 treated groups. The other experiments in this manuscript that did not involve mice used cellular samples, so randomization was not needed/not relevant.

Blinding

Blinding was not relevant to our study due to the nature of experiments employed. Blinding was not relevant/not needed because the outcomes of experiments were measured by a machine rather than a human (ie western blotting was imaged using a Biorad imager, mRNA expression was measured by a qPCR machine, apoptosis was measured using flow cytometry).

Reporting for specific materials, systems and methods

We require information from authors about some types of materials, experimental systems and methods used in many studies. Here, indicate whether each material, system or method listed is relevant to your study. If you are not sure if a list item applies to your research, read the appropriate section before selecting a response.

Materials & experimental systems

n/a	Involved in the study	
☐	☒	Antibodies
☐	☒	Eukaryotic cell lines
☒	☐	Palaeontology and archaeology
☐	☒	Animals and other organisms
☒	☐	Human research participants
☒	☐	Clinical data
☒	☐	Dual use research of concern

Methods

n/a	Involved in the study	
☒	☐	ChIP-seq
☒	☐	Flow cytometry
☒	☐	MRI-based neuroimaging

Antibodies

Antibodies used

The membrane was incubated with antibodies that target p53 (1:1000, Calbiochem Ab-6 clone DO-1), b-actin (1:1000, Sigma-Aldrich A5316 clone AC-74), phospho-S6K (T389) (1:1000, Cell Signaling Technology #9205), S6K (1:1000, Cell Signaling Technology #2708 Clone 49D7), phospho-S6 (S235/236) (1:1000, Cell Signaling Technology #2211), S6 (1:1000, Cell Signaling Technology #2217 5G10), phospho-4E-BP1 (Thr37/46) (1:1000, Cell Signaling Technology #2855), 4E-BP1 (1:1000, Cell Signaling Technology #2855), HIF-1α (1:1000 Novus Biologicals NB100-105), Cleaved PARP (1:1000, Cell Signaling Technology #5625), Caspase 3 (1:1000, Cell Signaling Technology #9662), Cleaved Caspase 3 (1:1000, Cell Signaling Technology #9661), PUMA (1:1000, Cell Signaling Technology #12450), RPL5 (1:1000, kindly provided by Dr. Siniša Volarević), RPL11 (1:1000, kindly provided by Dr. Siniša Volarević), phospho-MEK1/2 (Ser217/221) (1:500 Cell Signaling Technology #9121), MEK1/2 (1:500 Cell Signaling Technology #9122), phospho-ERK1/2 (Thr202/Tyr204) (1:500 Cell Signaling Technology #9101), ERK1/2 (Thr202/Tyr204) (1:500 Cell Signaling Technology #9102), phospho-eEF2 (Thr56) (1:1000 Cell Signaling Technology #2331), eEF2 (1:1000 Cell Signaling Technology #2332), eEF2K (1:1000 Cell Signaling Technology #3692), followed by incubation with horseradish peroxidase-conjugated secondary antibodies (1:5000, Sigma-Aldrich). Signals were detected using enhanced chemiluminescence detection reagents (Thermo Fisher Scientific) and images were acquired using a luminescent image analyzer (LAS3000, Fuji-Film, Japan).

Validation

All antibodies were validated by knocking down the target with a specific siRNA, or inducing the target with a drug (i.e. inducing p53 with CX-5461), or inhibiting the target with a drug (i.e. inhibiting mTORC1 phosphorylation with rapamycin)

Specifically, the following antibodies were validated by siRNA knockdown: p53 (Fig 4d), uL5 (Fig 4c), uL18 (Fig 4c), PUMA (Supplementary Fig 4a). The following antibodies were validated using drugs: phospho-S6K (Fig 5b), phospho-MEK (Fig 5c), phospho-ERK (Fig 5c).

Eukaryotic cell lines

Policy information about cell lines

Cell line source(s)	HCT116, A375, A549, U2OS, and MKN45, cells were purchased from the American Type Culture Collection (Manassas, VA, USA). HCT116 p53+/+ and p53−/− isogenic human colon cancer cells were kindly provided by Bert Vogelstein (Johns Hopkins University).
Authentication	Cell lines that were freshly purchased from the ATCC were authenticated by visual microscopy. Main cells such as HCT116, A375, and U2OS have very distinct morphologies that make them easy to identify. Also, since all cell lines are wild-type p53, we validated that these cells are p53 wild type using western blotting.
Mycoplasma contamination	The cell lines all tested negative for mycoplasma
Commonly misidentified lines (See ICLAC register)	No commonly misidentified cell lines were used.

Animals and other organisms

Policy information about studies involving animals; ARRIVE guidelines recommended for reporting animal research

Laboratory animals	Mice, nude strain, female, 8 weeks old, were used for tumor xenograft experiments.
Wild animals	*Provide details on animals observed in or captured in the field; report species, sex and age where possible. Describe how animals were caught and transported and what happened to captive animals after the study (if killed, explain why and describe method; if released, say where and when) OR state that the study did not involve wild animals.*
Field-collected samples	*For laboratory work with field-collected samples, describe all relevant parameters such as housing, maintenance, temperature, photoperiod and end-of-experiment protocol OR state that the study did not involve samples collected from the field.*
Ethics oversight	*Identify the organization(s) that approved or provided guidance on the study protocol, OR state that no ethical approval or guidance was required and explain why not.*

Note that full information on the approval of the study protocol must also be provided in the manuscript.
